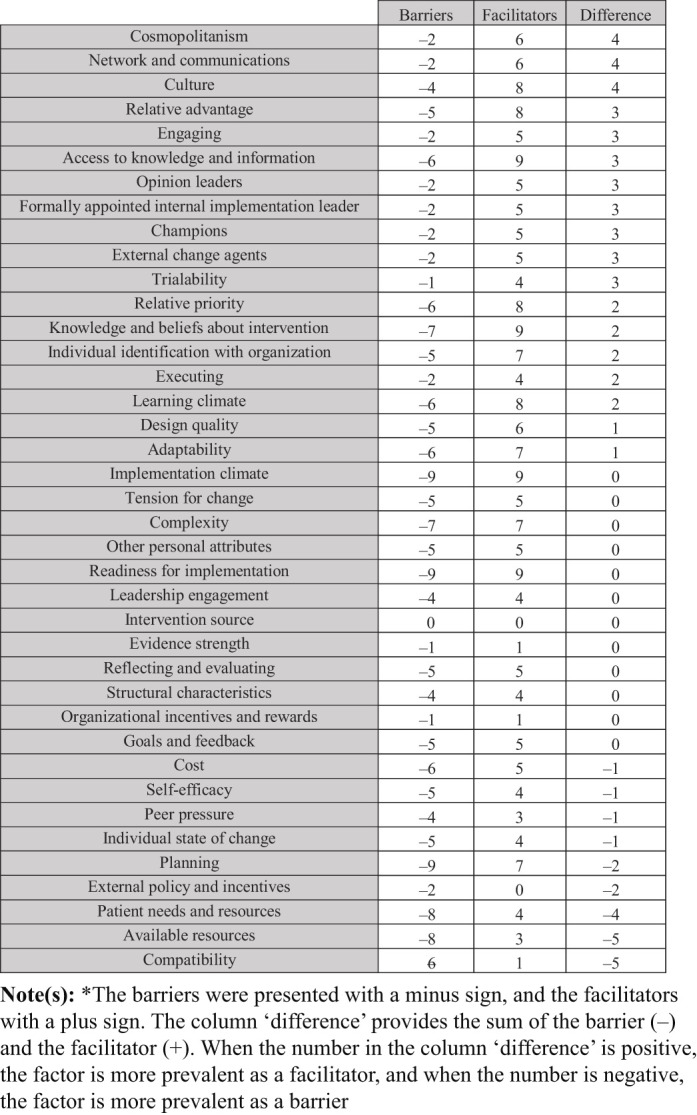# The barriers and facilitators of radical innovation implementation in secondary healthcare: a systematic review

**DOI:** 10.1108/JHOM-12-2020-0493

**Published:** 2021-12-16

**Authors:** Salina V. Thijssen, Maria J.G. Jacobs, Rachelle R. Swart, Luca Heising, Carol X.J. Ou, Cheryl Roumen

**Affiliations:** Department of Radiation Oncology (Maastro), GROW School for Oncology, Maastricht University Medical Centre+ , Maastricht, The Netherlands; Tilburg School of Economics and Management , Tilburg University , Tilburg, Netherlands

**Keywords:** Radical innovation, Barriers, Facilitators, Secondary healthcare, Implementation

## Abstract

**Purpose:**

This study aimed to identify the barriers and facilitators related to the implementation of radical innovations in secondary healthcare.

**Design/methodology/approach:**

A systematic review was conducted and presented in accordance with a PRISMA flowchart. The databases PubMed and Web of Science were searched for original publications in English between the 1st of January 2010 and 6th of November 2020. The level of radicalness was determined based on five characteristics of radical innovations. The level of evidence was classified according to the level of evidence scale of the University of Oxford. The Consolidated Framework for Implementation Research was used as a framework to classify the barriers and facilitators.

**Findings:**

Based on the inclusion and exclusion criteria, nine publications were included, concerning six technological, two organizational and one treatment innovation. The main barriers for radical innovation implementation in secondary healthcare were lack of human, material and financial resources, and lack of integration and organizational readiness. The main facilitators included a supportive culture, sufficient training, education and knowledge, and recognition of the expected added value.

**Originality/value:**

To our knowledge, this is the first systematic review examining the barriers and facilitators of radical innovation implementation in secondary healthcare. To ease radical innovation implementation, alternative performance systems may be helpful, including the following prerequisites: (1) Money, (2) Added value, (3) Timely knowledge and integration, (4) Culture, and (5) Human resources (MATCH). This study highlights the need for more high-level evidence studies in this area.

## Introduction

Radical innovations are well-recognized for their importance to enable renewal and long-term business growth ( Cake *et al.* , 2020 ). Research on knowledge intense firms illustrates that a difference can be detected between radical and incremental innovations ( Van Poucke, 2005 ). In contrast to incremental innovations, which focus on small but significant improvements, radical innovations are defined as: “creating dramatic change in technology, processes, products and/or services that considerably transforms existing markets and industries, or even gives rise to new ones” ( Miller *et al.* , 2005 ). Radical innovations comprise the following characteristics: (1) new engineering principles, (2) new scientific evidence, (3) new potential applications, (4) new markets, and, (5) new skills, competences, and knowledge different from those required to master the old processes ( Van Poucke, 2005 ). When considering radical innovation over incremental innovation, the latter may be preferred, e.g. in product development, as radical change may not be *desirable* (i.e. the current product may lead the market or have little competition), *feasible* (i.e. the technology for radical change may be immature for market release), or *profitable* (i.e. the current product may be top-grossing) ( Biskjaer *et al.* , 2019 ). In addition, radical innovations signify an extensive degree of uncertainty, instability, and unpredictability ( Bagno *et al.* , 2017 ). Yet, the opportunities of radical innovations can still outweigh the obstacles, as is demonstrated in the field of healthcare ( Bessant and Maher, 2009 ; Institute of Medicine (US), 2008 ). Examples of radical healthcare innovations include electrode implementation for spinal-cord injury ( Willyard, 2019 ), proton therapy for reduced toxicity ( Hu *et al.* , 2018 ), and nanotransfection to repair tissue ( Gallego-Perez *et al.* , 2017 ). Beyond the scope of healthcare, examples of radical innovations range from the LED light ( Phạm, 2011 ) and the self-driving car ( König and Neumayr, 2017 ), to cell-based tuna ( Reis *et al.* , 2021 ), which all contain three or more of the above-mentioned characteristics of a radical innovation. The positive effects of these advancements suggest that radical innovations are necessary in order to address and modernize the challenges faced in healthcare today ( Sehgal and Gupta, 2019 ). However, the implementation of incremental innovation has already proven to be difficult with regard to treatment, technological and organizational changes ( Jacobs *et al.* , 2016 ), let alone highly uncertain, instable and unpredictable radical innovations, where more than half of radical innovations are abandoned before completion ( Story *et al.* , 2011 ). For example, the slow implementation of proton therapy is resulting in major effects on patient outcomes, e.g. reduced quality of life ( Mohan and Grosshans, 2017 ), and overall survival rate ( Defraene *et al.* , 2020 ), even though it has been proven effective in cost and outcome for several cancer indications ( Ramaekers *et al.* , 2013 ). These challenges yield difficulties in simultaneously improving patient outcomes, patient safety, patient service as well as cost effectiveness ( Jacobs *et al.* , 2017 ).

Previous research beyond the scope of healthcare demonstrated that the most important barriers of radical innovation implementation are a restrictive mindset, lack of organizational competences, resistance or lack of support from actors, a restrictive external environment, insufficient resources (e.g. finance, skills, information and tools), and an unsupportive organizational culture ( Sandberg and Aarikka-Stenroos, 2014 ). Facilitators of radical innovation implementation beyond the scope of healthcare are the availability of human resources, the inclusion of important stakeholders, knowledge, supportive legislation and regulation, market opportunities, and product ease and readiness ( Dewar and Dutton, 1986 ; Moschos, 2016 ). Furthermore, some studies have indicated that exploitation forms an inhibition for radical innovation ( Benner and Tushman, 2002 ; March, 1991 ). In addition, a previous study revealed no considerable differences between barriers in product, system, solution, and technology innovations ( Sandberg and Aarikka-Stenroos, 2014 ). To date, however, no systematic literature review has been carried out to identify barriers and facilitators within secondary healthcare, possibly as research on implementation and radical innovations is a relatively new and neglected field in healthcare ( Peters *et al.* , 2013 ; Van Poucke, 2005 ). Nevertheless, the secondary healthcare sector is especially of importance due to their frontrunner role in applying complex, highly innovative products and processes for threatening diseases ( Weintraub and McKee, 2018 ).

This study will, therefore, aim to provide insights into the barriers and facilitators associated with the implementation of radical innovations, specifically in secondary healthcare (e.g. hospitals), and their relationship with the innovation type and the Consolidated Framework for Implementation Research (CFIR). We will investigate whether the barriers and facilitators of radical innovation implementation in secondary healthcare are in line with the barriers and facilitators of radical innovation implementation beyond the healthcare domain, and whether they are equivalent to the barriers and facilitators of incremental innovation implementation in healthcare. In a study developing a prediction model for successful implementation of incremental innovation in radiotherapy was found that sufficient and competent employees, awareness of the innovation, and desirability are significant predictive factors for timely implementation of incremental innovations ( Swart *et al.* , 2020 ). Furthermore, we want to investigate whether there is a difference in the barriers and facilitators between the type of innovation (e.g. technological, organizational and treatment), analogous to the scope outside of healthcare. This study can pave the way to close this knowledge gap, as well as enable generalizability and mutatis mutandis towards a common understanding and an approach to increase effectiveness and efficiency in future radical innovation implementation ( O'Connor, 2008 ).

## Research methodology

### Search strategy

**Figure 1:**
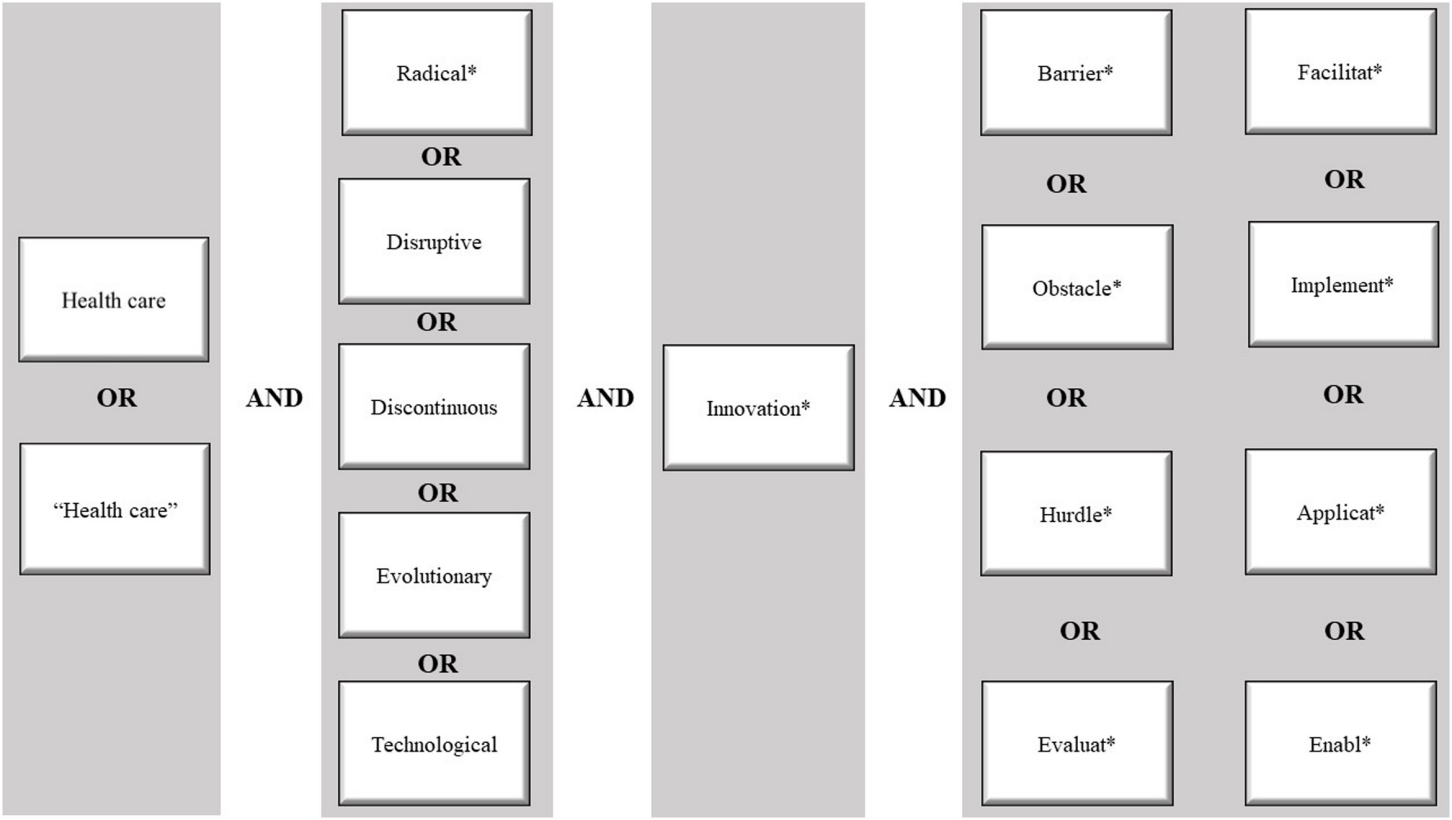
The PRISMA search strategy

We performed a systematic literature review to identify barriers and facilitators of the implementation of radical innovations in secondary healthcare, focusing on papers published between the 1st of January 2010 and 6th of November 2020. We executed four rounds of selection – database selection, keyword search, screening of the titles and abstracts, and screening of full texts. The databases PubMed and Web of Science (WoS) were searched for literature. The search strategy used in both databases is listed in Figure 1 . A Boolean search was performed, identifying search terms in the title and abstract for PubMed, and in the topic – this includes title, abstract and author keywords – for WoS. Variations of the search terms were included by using synonyms. After the term *innovation* , we used an asterisk (*) to assure that we included all radical innovations, including e.g. breakthrough innovation, revolutionary innovation, breakpoint innovation, or innovations involving a technological discontinuity. To specify, we did not regard a disruptive innovation as a radical innovation, as they both consist of distinctive characteristics. In disruptive innovations, new market entrants challenge the firms, whereas in a radical innovation a completely new market arises ( Hopp *et al.* , 2018 ). However, the term “disruptive” was still included in the search strategy, as these terms may have been used interchangeably. In addition, a requirement in our search strategy was that the articles should be written in English as translation services were not available. Furthermore, only Western countries were included to ensure generalizability.

### Study selection

Based on the search strategy, all relevant articles were downloaded into the data management tool Zotero. The systematic review was presented according to a PRISMA flowchart. To clarify, we only conducted a systematic review, where no statistical tests were included as in a meta-analysis ( Ahn and Kang, 2018 ). The duplicate articles were removed. Thereafter, the collected literature from the two databases was examined, based on the occurrence of the inclusion and exclusion criteria in the title and abstract of the papers ( Table 1 ). Uncertainties in data collection were discussed with another independent researcher until agreement was reached. The records were screened and eliminated based on the objectives of this systematic literature review and the results from cross-checking by another researcher.

All full text articles were classified on the radicalness of the innovations, based on the characteristics of radical innovations, covering: (1) new engineering principles, (2) new scientific evidence methods, (3) potential new applications, (4) new markets, and, (5) new competences or skills ( Van Poucke, 2005 ). These characteristics were used as a selection guideline to ensure only innovations perceived radical to organizations were included in this search. When the articles explicitly used the term “radical innovation” or “radical change”, the selected full text articles were included in the final review. Other eligible articles were included when at least three of the five characteristics of a radical innovation were present. As we only wanted to include papers with the majority of the characteristics of a radical innovation, the threshold of three out of five was chosen to omit the chance of selecting innovations that may be rather incremental instead of radical ( Van Poucke, 2005 ).

### Study analysis

**Figure 2:**
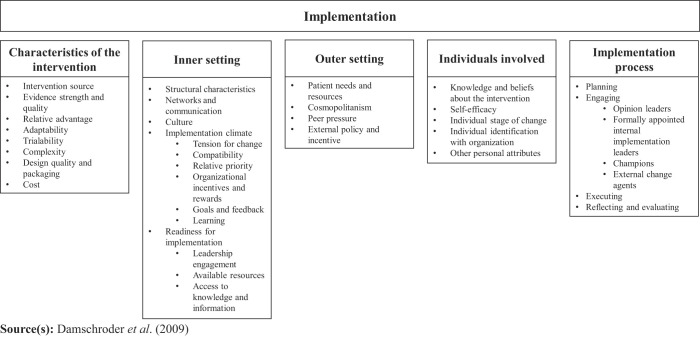
The consolidated framework for implementation research

We used the CFIR ( Damschroder *et al.* , 2009 ) for structuring the findings from the literature as it is designed to guide a scientific systematic assessment of multilevel implementation contexts ( Figure 2 ). The CFIR is a well operationalized, multi-level implementation determinant framework in healthcare and approaches potential influences on implementation. The CFIR is validated based on predictive and discriminant validity ( Assefa *et al.* , 2018 ). The framework comprises of 39 constructs (i.e. discrete theoretical concepts) across five domains, covering: (1) characteristics of the intervention, (2) inner setting, (3) outer setting, (4) individuals involved, and, (5) implementation process ( Damschroder *et al* ., 2009 , 2015 ). The CFIR has also been extensively used in past research to systematically identify and classify barriers and facilitators in implementation science, and is encouraged to be used in future implementation research designs ( Breimaier *et al.* , 2015 ; Harry *et al.* , 2019 ; Muddu *et al.* , 2020 ; Paulsen *et al.* , 2019 ; Powell *et al.* , 2014 ; Shade *et al.* , 2019 ). In addition, the type of innovation (technological, treatment and organizational) in each article was distinguished based on the definition as stated in literature ( Jacobs *et al.* , 2016 ). In our study, we handled the following definitions: (1) a technological innovation is the introduction of a new or improved technological process or method, (2) a treatment innovation is the introduction of treatments that are new or which account for a significant improvement in use or outcome, and (3) an organizational innovation is the introduction of an improved organizational structure, management method or system, whereby the use of knowledge, the quality of services or the efficiency of the workflow is improved ( Jacobs *et al.* , 2016 ).

Open coding was used to create labels and to identify overarching topics between the different articles. Thereafter, selective coding was used to classify the data from the literature into the five categories of the CFIR. In addition, the level of evidence scales of the University of Oxford (1: highest evidence – 5: lowest evidence) was used to identify the quality of evidence of the included articles in the final review. This scale was selected because it is one of the most accepted methods for classifying study quality, and takes the quality of the data into account, rather than only the study design ( Burns *et al.* , 2011 ; Graham *et al.* , 2004 ). In addition, even though our research does not specify at clinical research as such, this level of scale has been used and recommended in many different disciplines (e.g. prevention, diagnosis, prognosis, economic, and, decision analysis) ( Graham *et al.* , 2004 ; Lambin *et al.* , 2013 ; Richards, 2009 ). This scale allowed us to distinguish the included articles on their accuracy and internal validity, in order to draw an evidence-based conclusion. To assure external validity and the chance of transferability and generalizability, a detailed description was presented, which can be obtained and applied accordingly. Additionally, contrasting and disconfirming evidence was taken into account during the research to create informed subjectivity ( Holly *et al.* , 2017 ).

## Findings

### Literature search results

**Figure 3:**
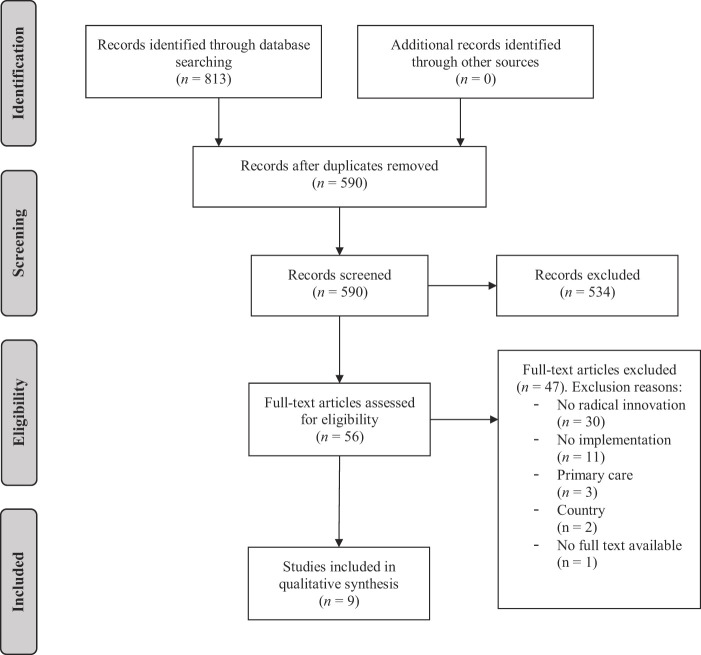
PRISMA flowchart on the data collection

A total of 813 publications were identified based on the keyword search, whereof 353 publications were retrieved from the database PubMed and 460 publications from WoS. After the duplicates were removed, a total of 590 publications remained. The residual publications were screened on title and abstract and were then selected dependent on the inclusion and exclusion criteria. This process resulted into the selection of 56 full-text publications. Thereof, 47 publications were excluded as they were not relevant to the aims and objectives of this literature study. The reasons for exclusion were: the innovation was not radical ( *n* = 30), the innovation did not have an implementation aspect ( *n* = 11), the innovation was implemented in primary care ( *n* = 3), the country where the innovation implementation took place was not in line with our criteria ( *n* = 2), or the full text was unavailable ( *n* = 1). In total, nine articles were eligible for inclusion in the final review. An overview of the PRISMA flowchart is presented in Figure 3 . Of the nine included articles, six included a technological innovation, two focused on an organizational innovation and one concerned a treatment innovation. Three of the nine articles were set in Norway. The other six studies were conducted in the Netherlands, Finland, Sweden, Belgium, Italy and the USA.

### Level of radicalness

All nine full-text articles were ranked on the five characteristics of radical innovations to evaluate the radicalness of the article's innovations and their eligibility for inclusion in the review ( Table 2 ). We regarded that the more radical and complex the innovation, the more characteristics of a radical innovation applied. Six out of the nine articles mentioned that the article contains a “radical innovation” or “radical change” and were therefore automatically eligible for inclusion. In three articles, the innovation was not explicitly mentioned as radical, but these articles were included because at least three of the five characteristics of a radical innovation were described in the papers. The studies of Melkas *et al.* (2020) , Van Bockhaven *et al.* (2017) and Mikhailova (2018) were distinguished as the most radical innovations, as the innovations mentioned in the articles scored on four of the five characteristics of a radical innovation. The innovations of Dugstad *et al.* (2019) , Coccia (2014) , and Söderholm *et al.* (2010) scored on three of the five characteristics. Two of the five characteristics were identified in Cramer *et al.* (2014) , Dugstad *et al.* (2020) and Tanniru *et al.* (2018) and were, thus, perceived the least radical innovations in this review.

### The main barriers and facilitators

The lack of material and human resources was identified as a barrier by eight of the nine studies and is, therefore, the most mentioned barrier in the implementation of radical innovations in secondary healthcare. One paper investigating a care system change ( Cramer *et al.* , 2014 ), did not report lack of human or material resources to be a barrier, but did mention financial resources as a constraint. In total, six studies reported lack of financial resources to be a barrier for radical innovation adoption, in which the type of innovation did not seem to make a difference. The lack of workflow integration and organizational readiness was recognized as the third most prevalent barrier, and also reported by six studies. The lack of training, education and knowledge about the innovation were reported by five studies as an important bottleneck.

The most mentioned facilitators among the articles investigating the implementation of radical innovations, concerned an open and supportive organizational culture, sufficient training, education and knowledge, as well as envisioned improvement in outcomes and added value, as documented by seven different papers respectively. Added value can be described as personal loss and gain, in which significant improvement in patient care or improved work ethics creates an incentive ( Melkas *et al.* , 2020 ). The three most mentioned barriers and facilitators are presented in Table 3 .

### Barriers and facilitators according to the type of innovation

An overview of all barriers and facilitators mentioned in the articles is summarized in Table 4 , where red represents a barrier, blue a facilitator, and purple both a barrier and facilitator. When comparing radical innovations to technological innovations, no substantial difference was found between the barriers and facilitators. The studies on technological innovations pointed out that the lack of material and human resources ( *n* = 6), lack of workflow integration and readiness ( *n* = 5), financial resources ( *n* = 4), insufficient staff motivation and commitment ( *n* = 4), beliefs about adverse outcomes and no recognition of added value ( *n* = 4), and an opposing organizational culture ( *n* = 4) were also barriers to radical innovation implementation of technologies, albeit to a lesser extent than the most mentioned barriers overall. In addition, a good workflow integration and organizational readiness, such as the use of operating protocols ( *n* = 5), a supportive organizational culture ( *n* = 5), and training, education and knowledge ( *n* = 5) appeared to be specifically facilitating factors for technological innovation implementation. In addition, four studies recognized sufficient financial resources, the inclusion of champions and the recognition of the expected outcomes and added value as facilitators.

Among the two articles on radical organizational innovation implementation, no similarities were observed in barriers. Supportive management and directors, an open and supportive organizational culture, sufficient training, education and knowledge, and improved outcomes and added value, were mentioned in both organizational articles as facilitators. The latter three were equal to the main facilitators within all articles. In contrast, Coccia *et al.* (2014) , being the only study on a radical treatment innovation, reported the least barriers regarding radical innovations, but highlighted mainly human and material resources as well as knowledge and training.

### Barriers and facilitators according to the CFIR

In this review, barriers and facilitators were classified according to the CFIR ( Damschroder *et al.* , 2009 ) to investigate whether all factors relevant for implementation, based on the well-established framework, were covered by the included studies ( Table 5 ). The most prevalent barriers based on the CFIR constructs were: (1) the lack of readiness for implementation ( *n* = 9), especially the lack of available resources ( *n* = 8), (2) the lack of planning ( *n* = 9), (3) an unstable implementation climate ( *n* = 9), and (4) the lack of patient needs and resources ( *n* = 8). The most mentioned facilitators were: (1) readiness for implementation ( *n* = 9), especially access to information ( *n* = 9), (2) access to knowledge and positive beliefs about the intervention ( *n* = 9), (3) a stable implementation climate ( *n* = 9), especially an open learning climate ( *n* = 8), (4) an open culture ( *n* = 8), (5) recognition of the relative advantage ( *n* = 8), and (6) recognition of relative priority ( *n* = 8).

We also analyzed which CFIR sub-constructs were most mentioned in the five main constructs. Concerning the characteristics of the intervention, the sub-constructs which were mentioned the most as a barrier were high complexity ( *n* = 7), high costs ( *n* = 6), and low adaptability ( *n* = 6), whereas the recognition of the relative advantage ( *n* = 8), low complexity ( *n* = 7), and high adaptability ( *n* = 7), were the main facilitators. Regarding the outer setting, the lack of tailoring to the patients' needs and resources was the most mentioned barrier ( *n* = 8), whereas adhering to cosmopolitanism ( *n* = 6) was the most mentioned facilitator. In the inner setting, the lack of readiness for implementation ( *n* = 9), the lack of available resources ( *n* = 9), and an unstable implementation climate ( *n* = 9), were the most frequently mentioned barriers. The main facilitators in the inner setting were the readiness for implementation ( *n* = 9), access to information and knowledge ( *n* = 9), a stable implementation climate ( *n* = 9), the recognition of relative priority ( *n* = 8), and an open learning climate and culture ( *n* = 8). When looking at the characteristics of the individuals involved in the implementation of radical innovations, access to knowledge and beliefs about the interventions was mentioned as a facilitator by all studies, and the lack thereof as a barrier by all except two studies. Regarding the implementation process, the lack of planning was mentioned as a barrier by all studies, and as a facilitator by seven studies when the importance of planning was recognized during implementation.

The intervention source was the only sub-category of the CFIR which was not touched upon in the literature with barriers nor facilitators. The pressure of external policies and lack of incentives were only identified as a barrier, but not as a facilitator. The three sub-categories least frequently represented in the barriers were trialability, organizational incentives and rewards, and evidence strength. The underrepresented facilitators in the CFIR constructs were peer pressure, organizational incentives and rewards, and evidence strength.

### The overlap between barriers and facilitators

**Figure 4:**
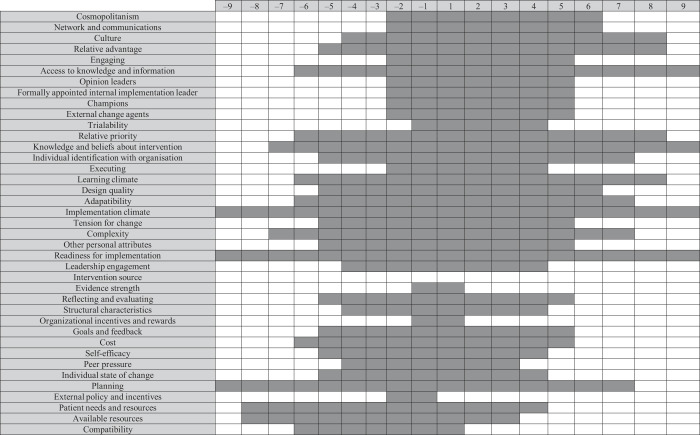
Visualization of the overlap between barriers and facilitators

We identified an overlap in barriers and facilitators in the implementation of radical innovations in secondary healthcare ( Table 6 and Figure 4 ). We found that the lack of available resources, patients' needs and resources, planning, individual state of change, self-efficacy, costs, peer pressure, compatibility, external policies and incentives were more prevalent as a barrier than as facilitator. The main facilitators which were more prevalent than the barriers are cosmopolitanism, culture, and networks and communication. Several factors were equally prevalent as barrier as facilitator, which, *inter alia* , were readiness for implementation, tension for change, and goals and feedback.

We also identified which factors were more prevalent as a barrier or facilitator, where we provided the sum of the barrier and facilitator under “difference”. This showed that the constructs of compatibility, available resources, patient needs and resources, and planning are the most prevalent barriers, as they were mentioned more often as a barrier than as a facilitator. The constructs of cosmopolitanism, networks and communication, and culture were the most prevalent facilitators. Amongst others, readiness for implementation, complexity and tension for change, were mentioned equally as much as facilitator as barrier.

## Discussion

**Figure 5:**
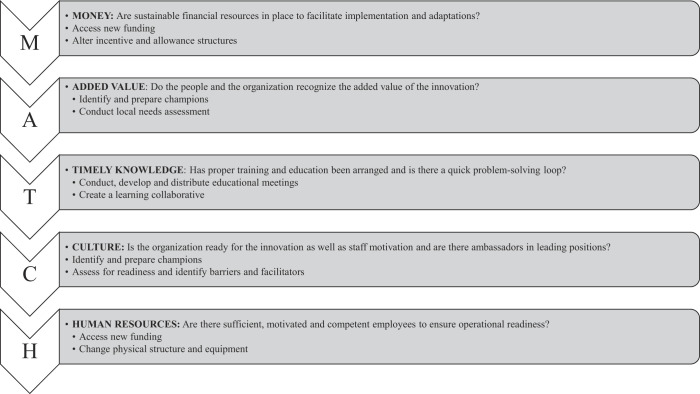
The MATCH checklist

Our results show that in secondary healthcare, the lack of human, material and financial resources, the lack of integration and lack of organizational readiness, are barriers to radical innovation implementation, whereas an open and supportive culture, sufficient training, education and knowledge, and the recognition of the added value, are facilitators to radical innovation implementation. We have, therefore, designed the MATCH checklist to provide guidance when implementing a radical innovation in secondary healthcare, referring to the following prerequisites: (1) **M** oney, (2) **A** dded value, (3) **T** imely knowledge and integration, (4) **C** ulture, and (5) **H** uman resources ( Figure 5 ). Based on the findings of this study, it was possible to provide an intervention list according to the Expert Recommendations for Implementing Change (ERIC) tool, as addition to the MATCH checklist, to achieve successful implementation outcomes ( Powell *et al.* , 2015 ). However, the additional ERIC interventions to overcome barriers for radical innovation are largely theoretical and will only work in practice if they will be made concrete in the context of the specific implementation. Nevertheless, it is important to pay attention to all these factors upfront to prevent hurdles in the implementation process. We found there was only a limited range of high-level evidence literature available, which besides radical innovations, also included the aspects of implementation barriers and facilitators. We have executed three ways of check-ups to ensure that no eligible literature was missed: (1) peer-reviewing, (2) computing different search strategies, (3) adhering to only two of the five characteristics of a radical innovation.

For the remainder part of the discussion, we will discuss the usefulness of the CFIR and overlap in barriers and facilitators, whereafter the main findings will be placed in context according to the three constructs of the CFIR in which the barriers and facilitators were most prevalent, namely (1) the characteristics of the innovation, (2) the inner setting, and (3) the characteristics of the individuals involved in implementation. Due to the low prevalence of barriers and facilitators in the outer setting and implementation process, we will not further discuss these CFIR constructs. For the characteristics of the innovation, we will discuss the level of radicalness and the different types of innovation. For the inner setting, we will discuss the organizational culture, implementation readiness, and the availability of resources, in which we will discuss how an ambidextrous organization may help to facilitate resources and innovation. For the individuals' characteristics, we will explore the influence of knowledge and beliefs. Lastly, we will discuss whether there are differences between incremental and/or radical innovations outside healthcare, as mentioned in the introduction.

### Main findings

Of all CFIR determinants, the lack of readiness and planning, and available resources (human, material and financial) were the most prevalent barriers to radical innovation implementation in secondary healthcare. In comparison to non-specific healthcare firms, the most prevalent barriers were a restrictive mindset, lack of organizational competences, resistance or lack of support from actors, a restrictive external environment, insufficient resources (e.g. finance, skills, information and tools), and an unsupportive organizational culture ( Sandberg and Aarikka-Stenroos, 2014 ). The (access to) knowledge and training, the learning climate, an open and supportive organizational culture, the relative advantage, priority, and beliefs were the main facilitators in our research, whereas beyond the scope of healthcare these were the availability of human resources, the inclusion of important stakeholders, access to knowledge, adequate legislation and regulation, good market opportunities, and technology or product ease and readiness ( Dewar and Dutton, 1986 ; Moschos, 2016 ). Based on these findings, we can conclude that the barriers and facilitators of radical innovations are not the same inside secondary healthcare as outside healthcare. Even though there is a slight overlap, certain barriers (e.g. lack of resources) are not as high of a priority beyond the scope of secondary healthcare. Interestingly, the main barrier we identified in radical innovation implementation in secondary healthcare, being lack of human and material resources, is not a barrier outside of healthcare. The facilitators of radical innovation implementation inside secondary healthcare overlap slightly with those of incremental innovations in radiotherapy; sufficient and competent employees, awareness of the innovation, and desirability ( Swart *et al.* , 2020 ). This is in contrast with findings in knowledge intensive firms, where a difference was established between incremental and radical innovations ( Van Poucke, 2005 ).

### The CFIR

The barriers and facilitators we found are in line with the constructs of the CFIR, which shows that the framework is useful in radical innovation implementation, despite the overlap in several constructs (e.g. planning vs organizational readiness, or implementation climate vs tension to change). Nonetheless, merely the CFIR construct of “available resources” is not sufficient for radical innovations since we identified the lack of human, material and financial resources as part of our most prevalent barriers. In addition, we found that the constructs of “inner setting” and “individuals involved” were of more influence in radical innovations, compared to the other three constructs.

### Characteristics of the intervention

#### Level of radicalness

Six articles adhered to three or more characteristics of a radical innovation. The three articles that only adhered to two characteristics, had in common that they specifically stated that the article included a radical innovation or radical change. In addition, the three articles used new skills and competences and implemented a system in their organization (i.e. an intelligent care system ( Tanniru *et al.* , 2018 ), a supply-driven long-term care system ( Cramer *et al.* , 2014 ), or a wireless nurse call system ( Dugstad *et al.* , 2020 )). Therefore, it is debatable whether these innovations are radical, as these innovations have several characteristics that are more in line with the characteristics of an incremental innovation.

#### Types of innovation

We were not able to draw a firm conclusion on the difference in barriers and facilitators between the types of innovation (i.e. technological, treatment and organizational) with regards to radical innovations, due to the small samples of the different innovation types. However, prior research illustrates that the facilitators for radical innovation implementations of technological innovations are; knowledge, ease of use, improved communication, staff motivation, and good organizational integration ( Schreiweis *et al.* , 2019 ). This is in line with our findings that IT infrastructure and mobile network instability – when inadequate – were the most frequently mentioned barriers to technology adaptation ( Dugstad *et al.* , 2019 ).

### Inner setting

#### Organizational resources

In our study, the human and material resources were frequently mentioned as barriers for implementation, specifically related to the need for orientation ( Melkas *et al.* , 2020 ), but also competition between departments ( Mikhailova, 2018 ), conflicts of interest, and lack of commitment ( Cramer *et al.* , 2014 ). This can be explained by the fact that the healthcare sector is under constraint to improve the quality and the efficiency of their services, while innovating at the same time ( Gastaldi *et al.* , 2018 ). Such a tension between maintaining the existing services and innovating may be mediated by ambidexterity. Ambidexterity is defined as the ability to both use and refine existing knowledge (exploitation) while creating new knowledge to overcome knowledge-gaps identified in the execution of the work (exploration) ( Foglia *et al.* , 2019 ; Turner *et al.* , 2013 ). While exploration focuses on experimenting, innovating, and looking for novelty ( Lavie *et al.* , 2010 ), exploitation focuses on recombining current knowledge, abilities to meet needs in changing times, and scarcity of resources ( Gastaldi *et al.* , 2018 ). It is currently unclear how innovation can best be nurtured; via exploration only, or by using exploitation and exploration simultaneously. As mentioned previously, some studies have indicated that exploitation forms an inhibition for radical innovation ( Benner and Tushman, 2002 ; March, 1991 ), although there are also studies that support coexistence of exploitation and exploration as long as there is a supportive business context ( Gibson and Birkinshaw, 2004 ; Katila and Ahuja, 2002 ). The latter is also referred to as the ambidextrous organization and is the dominant point of view in literature ( O'Reilly and Tushman, 2004 ). As our study indicates, ambidexterity-related issues may be particularly relevant for the design and finance structures that foster both attending of patients and using digital health care systems ( Soderholm and Sonnenwald, 2010 ), as well as allocating time and resources to daily activities and innovation implementation ( Dugstad *et al.* , 2019 ).

#### Organizational culture

Within an innovative organization, there may be a seemingly conflicting nature of exploitation of daily activities vs exploration of innovative leads, and this may be partly resolved by individual characteristics and attitudes and other cultural elements. In hospital contexts it has been demonstrated that lead physicians' leadership style as well as organizational creativity plays a key role in materializing ambidextrous behaviors ( Foglia *et al.* , 2019 ). Also, learning behavior has been positively associated with team outcome and innovation ( Lavie *et al.* , 2010 ; Miron-Spektor *et al.* , 2011 ). A recent study beyond healthcare showed that a high level of learning behavior results in complementary exploitation and exploration, whereas a low learning behavior causes a competition between them ( Blank and Naveh, 2018 ). Interestingly, we found that a high level of learning behavior could facilitate exploration and radical innovation, e.g. by an increased ability in multitasking ( Soderholm and Sonnenwald, 2010 ), while also contributing to exploitative operations. In the manufacturing industry however, such a symbiotic nature does not seem to work for radical innovation ( Lennerts *et al.* , 2020 ), as in the manufacturing industry radical innovation seems to rely solely on exploration. In contrast, incremental innovations can flourish by maintaining a balance between exploitation and exploration, which can be explained by the size and impact of the innovation ( Lennerts *et al.* , 2020 ).

Whereas innovation barriers do not seem to differ with varying degrees of novelty, the size of the organization may have an influence, which is illustrated by some studies, by for example the dependence of SMEs ( Sandberg and Aarikka-Stenroos, 2014 ) and government organizations ( Cramer *et al.* , 2014 ) on external financial resources to implement and sustain innovations. For larger organizations, however, the internal resistance may play a more central role due to lack of innovative infrastructures ( Coccia, 2014 ) or the mobilization across different departments or sectors ( Van Bockhaven and Matthyssens, 2017 ). In a recent review, radical innovation was investigated along performance management networks and revealed that radical innovation express alternative performance management features, including a recursive, e.g. agile way of working, as well as openness and unintended performance ( Gomes *et al.* , 2019 ). Ambidextrous managers should be considerate about the organizational needs and allow free-thinking, and at the same time maintain objectivity to make difficult trade-offs ( O'Reilly and Tushman, 2004 ).

#### Implementation climate and readiness

The readiness of an organization according to the CFIR ( Damschroder *et al.* , 2015 ) is in line with our study, and is represented by the capabilities for problem solving, technological infrastructure, leadership, learning new knowledge externally, sharing knowledge internally and relational capacity. Similarly, prior healthcare research shows that strategies to improve the organizational readiness include the development of incentives, improved knowledge, inter-organizational collaboration and the development of an innovation structure ( Williams and Weber-Jahnke, 2010 ). However, the use of incentives is partial in this review, where the lack of incentives could reduce the drive of healthcare providers to commit more time ( Soderholm and Sonnenwald, 2010 ), or healthcare professionals still resisted to commit to the innovation, even after monetary incentives were presented ( Van Bockhaven and Matthyssens, 2017 ).

Our study shows that training in radical innovation implementation should entail tailored information in order to apply it into the staff's routines. Furthermore, practical handling of the new innovation by learning from other professionals and programs that have similar interest and visions, is found to be useful ( Cramer *et al.* , 2014 ; Dugstad *et al* ., 2019 , 2020 ; Soderholm and Sonnenwald, 2010 ). In both secondary and primary healthcare, external partnerships and inter-organizational collaborations do not only assist radical healthcare innovation implementation in a social exchange, but also in securing resources and the development of new skills and evidence ( Barnett *et al.* , 2011 ; Mikhailova, 2018 ). In accordance with beyond the scope of healthcare, we found that openness to change and learning, including social support from colleagues and management, can be dependent on the culture or structure of an organization ( Tiberius *et al.* , 2020 ). A radical innovation management checklist (i.e. the MATCH checklist) could provide managers with a useful tool for radical innovation implementation. This would also assist in identifying organizational gaps, including the internal climate, management teams, emotional capacity of staff and available human resources ( Tiberius *et al.* , 2020 ).

It is worthy to highlight that the COVID-19 crisis showed the importance of radical change in transforming healthcare, due to a tremendous shift in both the market and healthcare services ( McCausland, 2020 ), where the SARS-CoV-2 vaccine is surpassing the average licensure and approval pace in fivefold ( Alkandari *et al.* , 2021 ). The pandemic also confirmed our findings that material, human and financial healthcare resources, and the integration of new protocols for staff are crucial factors in radical innovation implementation ( Anoushiravani *et al.* , 2020 ; Iyengar *et al.* , 2020 ; Satiani *et al.* , 2020 ). Previous studies show that radical innovation implementation can be enforced and driven by a specific healthcare crisis ( Alkandari *et al.* , 2021 ; Anoushiravani *et al.* , 2020 ). However, we should strive for such efficient adoption, also without the underlying pressure of an extensive crisis.

### Characteristics of individuals

#### Knowledge and beliefs

The individuals' attitudes toward, and value placed on the innovation, as well as the knowledge of rightness related to the intervention, also influences the implementation of innovations in healthcare, and radical innovations beyond healthcare ( Damschroder *et al.* , 2009 ; Tellis *et al.* , 2009 ). This study shows that, even though a radical innovation consists of new evidence methods, evidence and trials as such are not necessarily a prerequisite for care providers before willing to use the innovation ( Soderholm and Sonnenwald, 2010 ). This is dissimilar to the implementation of disruptive innovations in healthcare services ( Barnett *et al.* , 2011 ). However, the need for quantitative evidence is only a requirement for certain professional groups in healthcare ( Barnett *et al.* , 2011 ). In line with our research, a previous study in social care reports found that hard scientific evidence is not related in a linear way to innovation adoption ( Denis-Lalonde *et al.* , 2019 ). Thus, while randomized clinical trials are the standard for acquiring clinical evidence of efficacy and benefits of new treatments, they do not always seem a necessity in radical innovation implementation, as the study approach depends on the device itself ( KNAW, 2014 ). In new medical devices, there is often an intricate interplay between the technical complexities of the device, its user, and user learning curve effects, compromising intervention adherence and the possibility of randomized allocation ( KNAW, 2014 ).

As in line with the outcomes of our research, a negative attitude toward the innovation often results in resistance to change and an oppression to taking risks ( Sandberg and Aarikka-Stenroos, 2014 ). This barrier of a restrictive mindset is also recognized in reviews on innovation barriers in general ( Hölzl and Janger, 2012 ). In medical equipment technology, users with a unique set of characteristics, including high motivation toward new solutions, and who are embedded in a very supportive context, can contribute largely in radical innovations, which is in line with our findings ( Barnett *et al.* , 2011 ; Lettl *et al.* , 2006 ). This also relates to our findings of the integration of champions, influential mediators or ambassadors, through media attention and academic publications, who ensure successful organizational implementation and spread within the market ( Barnett *et al.* , 2011 ; Mikhailova, 2018 ).

### Contributions and limitations

The strength of this research is that it is the only systematic literature review performed on the barriers and facilitators of radical innovation implementation in secondary healthcare. With its highest level of evidence methodology, this study is highly reliable in the scientific evidence produced and valid in any context in secondary healthcare in the Western World. However, the terms radical, disruptive, discontinuous and evolutionary have been used in literature interchangeably and not consequently. Therefore, radical innovations listed by the product or service name only, and not in combination with one of the above terms, may have been left out unintentionally. As there are only few studies available on this topic in healthcare, it limited the ability to draw firm conclusions. Therefore, we urge high-level research in the area of radical innovation implementation in healthcare, so that patients can benefit from innovations more rapidly. To investigate why certain determinants are facilitators or barriers, we suggest multiple case study investigations with interviews and focus groups to acquire more insight and qualitative information. In addition, as there appears to be more overlap with incremental innovations inside healthcare, in comparison to radical innovations outside healthcare, a contextual analysis could be useful. Furthermore, it would be helpful to gain insight into the effectiveness of radical innovations, by reporting on the (non-)achievement of predefined targets. In addition, the magnitude and statistical significance of determinants could help the development of a prediction model, as is already present for incremental innovations ( Swart *et al.* , 2020 ). We have now focused on the prevalence of barriers and facilitators. However, the prevalence of a barrier or facilitator, does not necessarily mean it is the most influential one. Therefore, additional qualitative research can aid in determining the most pressing barrier or facilitator in a specific context where a radical innovation is implemented.

### Conclusion

In secondary healthcare, the lack of human, material and financial resources and, the lack of workflow integration and organizational readiness are barriers inhibiting radical innovation implementation, while an open and supportive culture, recognition of the added value and sufficient knowledge will help ease the implementation. There appears to be more overlap with incremental innovations inside healthcare, in comparison to radical innovations outside healthcare. Therefore, it can be argued that the context of innovation implementation is more influential than the radicalness of the innovation. We suggest the use of the MATCH checklist, as developed by this study, when implementing a radical innovation in secondary healthcare. This concludes that, if the relative advantage of a radical innovation is clear, the right culture and knowledge in the organization is adapted, and resources are available, radical innovation implementation is expected to have the highest chance of success in secondary healthcare.

## Figures and Tables

**Table 1:** The inclusion and exclusion criteria

Inclusion criteria	Exclusion criteria
Available in full-text	No full-text available
Written in English	Not written in English
Radical innovation, based on the five criteria stated in the full-text of the publication	Not a radical innovation
Western countries: USA, Luxembourg, England, Italy, Portugal, Netherlands, New Zealand, Canada, Australia, Wales, France, Czech Republic, Slovenia, Sweden, North Ireland, Spain, Bulgaria, Germany, Estonia, Norway, Switzerland, Lithuania, Belgium, Finland, Ireland, Scotland, Slovakia, Austria, Denmark, Hungary, Poland or Latvia	Non-Western countries
In the secondary healthcare setting	Not generalizable: home-based programs, primary healthcare setting and too much focus on the medical aspect
An element of implementation	No element of implementation
Article, review, book chapter or proceedings paper	Sources other than article, review, book chapter or proceedings paper

**Table 2:** Radicalness of the innovations

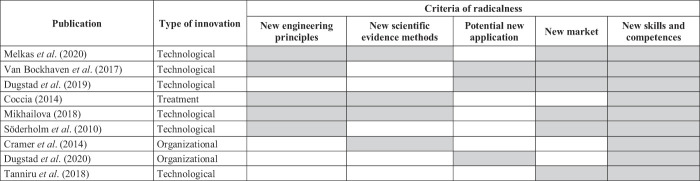

**Table 3:** Main barriers and facilitators

Barriers	Facilitators
Lack of material and human resources ( *n* = 8)	Open and supportive organizational culture ( *n* = 7)
Lack of financial resources ( *n* = 6)	Sufficient training, education and knowledge ( *n* = 7)
Lack of integration and readiness ( *n* = 6)	Outcomes and added value recognized ( *n* = 7)

**Table 4:** Barriers and facilitators according to the type of innovation

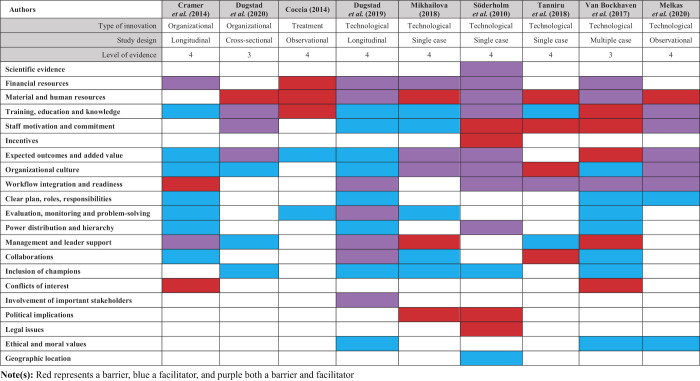

**Table 5:** Barriers and facilitators according to the CFIR

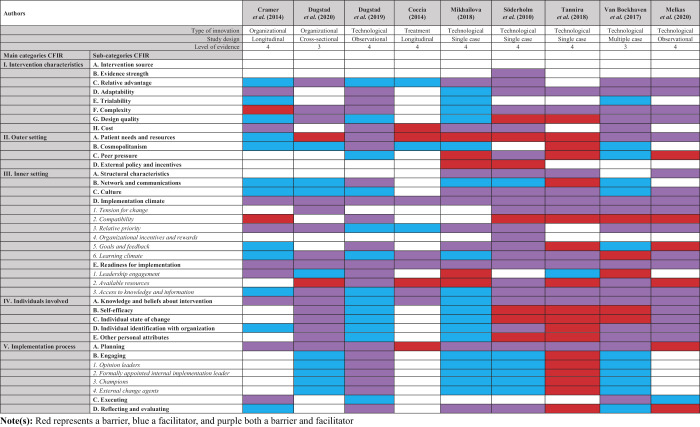

**Table 6:** Overlap in barriers and facilitators